# Editorial: Regulation of neuroinflammation by multiorgan network

**DOI:** 10.3389/fnagi.2022.1047113

**Published:** 2022-09-30

**Authors:** Yoshihisa Koyama, Takahide Itokazu, Tsuyoshi Hattori, Hironori Takamura

**Affiliations:** ^1^Department of Neuroscience and Cell Biology, Osaka University Graduate School of Medicine, Osaka, Japan; ^2^Addiction Research Unit, Osaka Psychiatric Research Center, Osaka Psychiatric Medical Center, Osaka, Japan; ^3^Department of Neuro-Medical Science, Graduate School of Medicine, Osaka University, Osaka, Japan; ^4^Department of Molecular Neuroscience, Graduate School of Medicine, Osaka University, Osaka, Japan; ^5^Department of Neuroanatomy, Graduate School of Medical Sciences, Kanazawa University, Ishikawa, Japan; ^6^Department of Child Development and Molecular Brain Science, United Graduate School of Child Development, Osaka University, Osaka, Japan

**Keywords:** neuroinflammation, blood-brain barrier, brain-organ axis, neurodegenerative diseases, psychiatric diseases

Neuroinflammation is a biological response initiated by tissue injury or infection in the central nervous system (CNS) to eliminate pathogenic components and induce tissue remodeling. However, insufficient control of neuroinflammation leads to the progression of many neurological conditions such as multiple sclerosis, traumatic brain injury, and Alzheimer's disease (Akiyama et al., 2000; Lucas et al., 2006; Frischer et al., 2009). Glial cells, including microglia and astrocytes, are involved in the immune response in the CNS and play important roles in the development of neuroinflammation. It is well documented that sustained inflammatory responses cause the release of harmful mediators such as cytokines and chemokines from activated glial cells, and further affect neuronal cells by triggering neurodegeneration (Liu et al., 2011; Lian et al., 2016; Norden et al., 2016; Jo et al., 2017). Neuroinflammation also is involved in the pathology of psychiatric diseases such as depression. Neuroinflammation such as increased cytokines in the brain affect kynurenine pathway and HPA axis, which can cause impairment of hippocampal neurogenesis (Troubat et al., 2021).

On the other hand, neuroinflammation is closely related with systemic immune systems outside the brain. Patients with inflammatory bowel diseases such as ulcerative colitis have a high prevalence of depression and anxiety, because of bidirectional communication *via* gut-brain axis which regulates inflammatory response and immune homeostasis (Agirman et al., 2021; Barberio et al., 2021). Furthermore, fetal exposure to a maternal infection while hospitalized increased risk for autism and depression during the child's life (Al-Haddad et al., 2019). These studies suggest close relationship in pathologies between systemic inflammation and mental diseases.

In the review article entitled “Inflammation from Peripheral Organs to the Brain: How Does Systemic Inflammation Cause Neuroinflammation?”, Sun et al. provide an overview of the relationship between neuroinflammation and systemic inflammation. First, the authors summarize the evidence of brain inflammation caused by peripheral inflammation. Then, discussion on several possible pathways of peripheral inflammatory mediators to enter the central nervous system are provided. Of note, they also discuss the cause of region-specific susceptibility of the brain to systemic inflammation. This review provides interesting perspective as well as basic information for future research in this field.

Miyata et al., review recent literature and discuss possible roles of inflammation for stress-induced major depressive disorder (MDD). Inflammation and immune response in the brain and peripheral tissues are associated with onset of MDDs. Chronic stress can induce microglial activation through affecting various molecules such as glucocorticoid, noradrenaline, and cGAS-STING pathway. Moreover, chronic stress-induced neuroinflammation can affect depressive behaviors by causing functional and morphological abnormalities of oligodendrocytes and decrease of astrocytic cell density.

Ito et al., review the recent reports and discuss the pathophysiology and clinical characteristics of septic encephalopathy from the perspective of region-specific inflammation. Endothelial cell degeneration, increased BBB permeability and loss of tight junction proteins may be the underlying mechanisms of septic encephalopathy. However, it remains unclear why some brain regions develop inflammation and degeneration, and the factors that determine the persistence of neuroinflammation. Interestingly, decrease in regional cerebral blood flow and decreased glucose uptake differed across brain regions, which may influence region-specific inflammatory vulnerability.

Ya et al., revealed that rats fed a high-fat diet with carotid artery injury exhibited impaired spatial learning and memory through systemic inflammation, cerebral microvascular endothelial activity, and abnormal lipid metabolism. Furthermore, they also proved that these abnormalities were ameliorated by curcumin, an antioxidant. Nervous system, endocrine system and immune system interact with each other, and their imbalance is greatly involved in the pathogenesis of cerebrovascular disease. The importance of this balance is also clear from the fact that the regulation of cerebral microvascular inflammatory response and lipid metabolism by curcumin administration regulates neuroendocrine immune changes. It is very interesting that memory impairment also occurs in systemic inflammation caused by dyslipidemia.

Accumulating evidence suggests that inflammatory processes are critically involved in most of the neurological disorders including neurodegenerative diseases such as Parkinson's disease as well as neuroinflammatory diseases such as multiple sclerosis. Although the role of neuroinflammation in these conditions are intensively investigated, whether and how neuroinflammation is regulated by systemic inflammation is still unclear due to the complexity of biological reaction brought by multi-organ interaction. In addition, in the LPS intraperitoneal injection model, neuroinflammation is observed in the hippocampus and prefrontal cortex in the chronic phase, but in the hypothalamus in the acute phase (Gatti and Bartfai, 1993; Bossù et al., 2012). The mechanism by which the area of temporal neuroinflammation changes over time is also not well understood. Therefore, understanding the detailed mechanism of neuroinflammation from the periphery and elucidating the determinants of inflammatory vulnerability in brain regions will not only protect the brain from peripheral inflammation, but also lead to complete cure of peripheral inflammation. We hope that this e-book will contribute to this research field.

## Author contributions

YK, TI, TH, and HT wrote the paper, discussed the findings, and commented on this manuscript. All authors contributed to the article and approved the submitted version.

## Funding

This work was supported by JST SPRING (Grant Number JPMJSP2138).

## Conflict of interest

The authors declare that the research was conducted in the absence of any commercial or financial relationships that could be construed as a potential conflict of interest.

## Publisher's note

All claims expressed in this article are solely those of the authors and do not necessarily represent those of their affiliated organizations, or those of the publisher, the editors and the reviewers. Any product that may be evaluated in this article, or claim that may be made by its manufacturer, is not guaranteed or endorsed by the publisher.
